# Dr. W. E. Magill

**Published:** 1896-07

**Authors:** 


### Obituary.

### DR. W. E. MAGILL.

Died May 26, 1896, at Erie, Pa., of apoplexy, Dr. W. E. Magill. The death of this prominent and universally esteemed member of the dental profession occurred while apparently in good health and at work in his laboratory. He was born at Meadville, Pa., May 29, 1825, and entered as a student of dentistry in Dr. A. B. Robbins's office in Meadville in 1844. He commenced the practice of dentistry in the same place in 1848, and removed to Erie, Pa., two years later, where he was in continuous practice up to the time of his death.

Dr. Magill was a member of the Pennsylvania State Dental Society from its first organization, and at one time filled the office of president. He also was president of the Lake Erie Dental Society. For many years he has been an active and influential member of the Pennsylvania State Dental Examining Board.

The death of Dr. Magill leaves a vacancy in the ranks of the active workers in dentistry in Pennsylvania that will not soon be filled. His very clear, logical mind made him a power in the State and local organizations, and his earnest devotion to the best interests of his calling, together with his kindly feeling for his coworkers, made him always welcome in professional circles.

To the writer this death is a personal loss. Mingling with Dr. Magill for many years in the arena of professional society work, he had learned to appreciate him not only for his marked ability, but as a friend and valued counsellor.

The dental profession has lost in the past few years many of its brilliant men. While this must continue to be true in this ever-changing life, we never become reconciled to it, for seemingly many links have been broken in the chain never to be replaced.

Dr. Magill was married to Louisa Jones at Erie, January 15, 1857. He leaves a wife and three sons living,—Dr. W. J. Magill, of Erie; Edward S. and Charles J., of Chicago, and Louis J., of the United States navy.

